# Robust detection of immune transcripts in FFPE samples using targeted RNA sequencing

**DOI:** 10.18632/oncotarget.13691

**Published:** 2016-11-29

**Authors:** Benjamin E Paluch, Sean T Glenn, Jeffrey M Conroy, Antonios Papanicolau-Sengos, Wiam Bshara, Angela R Omilian, Elizabeth Brese, Mary Nesline, Blake Burgher, Jonathan Andreas, Kunle Odunsi, Kevin Eng, Ji He, Maochun Qin, Mark Gardner, Lorenzo Galluzzi, Carl D Morrison

**Affiliations:** ^1^ Omniseq LLC, Buffalo, NY 14203, USA; ^2^ Department of Cancer Genetics, Roswell Park Cancer Institute, Buffalo, NY 14263, USA; ^3^ Center for Personalized Medicine, Roswell Park Cancer Institute, Buffalo, NY 14263, USA; ^4^ Department of Pathology, Roswell Park Cancer Institute, Buffalo, NY 14263, USA; ^5^ Department of Gynecologic Oncology, Center for Immunotherapy, Department of Immunology, Roswell Park Cancer Institute, Buffalo, NY 14263, USA; ^6^ Department of Biostatistics, Roswell Park Cancer Institute, Buffalo, NY 14263, USA; ^7^ Department of Radiation Oncology, Weill Cornell Medical College, New York, NY 10065, USA; ^8^ Equipe 11 labellisée Ligue contre le Cancer, Centre de Recherche des Cordeliers, 75006 Paris, France; ^9^ INSERM, U1138, 75006 Paris, France; ^10^ Université Paris Descartes/Paris V, Sorbonne Paris Cité, 75006 Paris, France; ^11^ Université Pierre et Marie Curie/Paris VI, 75006 Paris, France; ^12^ Gustave Roussy Cancer Campus, 94805 Villejuif, France

**Keywords:** cancer immunotherapy, CD8+ cytotoxic T lymphocytes, nivolumab, NY-ESO-1, PD-L1

## Abstract

Current criteria for identifying cancer patients suitable for immunotherapy with immune checkpoint blockers (ICBs) are subjective and prone to misinterpretation, as they mainly rely on the visual assessment of CD274 (best known as PD-L1) expression levels by immunohistochemistry (IHC). To address this issue, we developed a RNA sequencing (RNAseq)-based approach that specifically measures the abundance of immune transcripts in formalin-fixed paraffin embedded (FFPE) specimens. Besides exhibiting superior sensitivity as compared to whole transcriptome RNAseq, our assay requires little starting material, implying that it is compatible with RNA degradation normally caused by formalin. Here, we demonstrate that a targeted RNAseq panel reliably profiles mRNA expression levels in FFPE samples from a cohort of ovarian carcinoma patients. The expression profile of immune transcripts as measured by targeted RNAseq in FFPE *versus* freshly frozen (FF) samples from the same tumor was highly concordant, in spite of the RNA quality issues associated with formalin fixation. Moreover, the results of targeted RNAseq on FFPE specimens exhibited a robust correlation with mRNA expression levels as measured on the same samples by quantitative RT-PCR, as well as with protein abundance as determined by IHC. These findings demonstrate that RNAseq profiling on archival FFPE tissues can be used reliably in studies assessing the efficacy of cancer immunotherapy.

## INTRODUCTION

During the past few years, no less than four distinct monoclonal antibodies (mAbs) that interrupt immunological checkpoints, so-called immune checkpoint blockers (ICBs) have been approved by the US Food and Drug Administration (FDA) for use in cancer patients as standalone immunotherapeutic regimens or combined with other drugs [1]. These agents include the cytotoxic T lymphocyte-associated protein 4 (CTLA4)-specific mAb ipilimumab (Yervoy), which is licensed for the treatment of melanoma [2–4]; two mAbs targeting programmed cell death 1 (PCDC1; best known as PD-1), namely nivolumab (Opdivo) and pembrolizumab (Keytruda), which are approved for use in patients with melanoma, head and neck squamous cell carcinoma, non-small cell lung carcinoma (NSCLC) and Hodgkin's lymphoma [5–14]; and atezolizumab (Tecentriq), a mAb targeting CD274 (best known as PD-L1) recently approved for use in bladder carcinoma patients [15, 16]. ICBs can mediate robust clinical effects as they release immune effectors from cancer-driven immunosuppression, hence activating novel or reactivating existing tumor-targeting immune responses [17]. However, only a limited fraction of patients (generally <30%) benefit from ICBs as standalone immunotherapeutic agents [1]. Moreover, it is estimated that the total market for ICBs may reach 7 billion US by 2020 [18]. Thus, there are both clinical and economical challenges associated with the growing use of ICBs, calling for the development of cost-effective and reliable selection procedures.

Currently, the immunohistochemical assessment of PD-L1 expression level on formalin-fixed paraffin-embedded (FFPE) specimens is the only test employed in the clinic to guide the use of ICBs, and is an approved companion diagnostic for NSCLC patients considered for pembrolizumab treatment [19, 20]. Other potential indicators of response to checkpoint inhibition include mutational burden [21–23] and tumor-infiltrating lymphocyte (TIL) abundance [24–26], but both lack sensitivity and specificity as single biomarkers. An alternative approach to predicting clinical response to ICBs involves digital gene expression analysis by RNA next generation sequencing (NGS). While this methodology works well on fresh frozen (FF) samples, it has demonstrated suboptimal performance on more readily available archival FFPE specimens [27, 28]. Thus, there is no test available today to reliably predict whether a cancer patient will respond to immunotherapy with ICBs.

To begin to address this major gap in clinical care, we applied targeted amplicon-based RNA sequencing (RNAseq) to a panel of 395 transcripts related to T-cell receptor signaling (TCRS), tumor infiltration by immune cells, and other immunological functions that are key for anticancer immunosurveillance. RNAseq is particularly adept at detecting poorly represented transcripts. We optimized our assay for RNA isolated from FFPE samples, which suffer from RNA degradation as a result of formalin fixation. We are currently focusing on a subset of these genes to generate a focused RNAseq panel (which we named Immune Advance) that predict clinical response to ICBs.

Here, we present data demonstrating that the Immune Advance assay on FFPE samples is associated with a low failure rate and produces gene expression profiles that are highly concordant with those obtained on FF specimens. Moreover, we report that the expression levels of three prototypic biomarkers, namely CTAG1B (a tumor-associated antigen best known as NY-ESO-1) [29], CD8 (a biomarker of cytotoxic T lymphocytes) [30, 31], and PD-L1 (see above) [32], measured by RNAseq, quantitative RT-PCR (qRT-PCR) and immunohistochemistry (IHC) exhibit high levels of correlation. Thus, the Immune Advance assay can accurately profile gene expression in FFPE samples as an instrument to predict clinical response to ICBs.

## RESULTS

### Reproducibility of the immune advance assay

**Figure 1:**
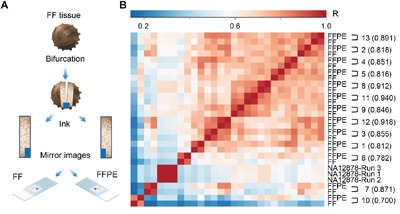
Immune Advance assay performance on FFPE versus FF samples **A**. Samples from 14 ovarian cancer patients were halved to generate a series of matched fresh frozen (FF) and formalin-fixed paraffin embedded (FFPE) specimens, which were serially sectioned, and processed for further analysis. **B**. Targeted RNAseq on a panel of immunological transcripts was performed on 13 samples pairs that passed quality control upon RNA extraction, as well as on control sample NA12878 in triplicate runs. Each FFPE/FF sample pair demonstrated unique correlation distinct from all other specimens. The matrix depict inter-sample correlation based on Pearson correlation coefficient (R). R^2^ are indicated for each sample pair in parentheses.

RNA was extracted on matched FFPE and FF sections from 14 ovarian cancer specimens (Figure 1A), and analyzed across three RNAseq runs. The average mean read length was 112 bp and the percentage of aligned bases was 96% (Supplementary Table S1). All samples had a minimum mapped reads of 2,554,065 with the exception of one sample, 14-FFPE, with 2,354 mapped reads (Supplementary Table S2), which failed our internal quality control. On average, we achieved 4,432,474 mapped reads (after excluding sample 14-FFPE), which represents a sufficient depth for digital gene expression profiling of 395 genes. Likewise, 89.55% mapped reads were on target, which is consistent with best RNAseq practice [33]. Overall 27/28 samples passed quality control, indicating 100% and 93% assay robustness for FF and FFPE samples, respectively. Normalized reads per million (nRPM) values derived from each FFPE/FF sample pair were correlated using the Pearson method. The mean, minimum, and maximum Pearson correlation coefficients obtained from 13 paired FFPE/FF samples were 0.920, 0.837, and 0.969, respectively (Figure 1B and Supplementary Figure S1).

### Clinical validity

**Figure 2:**
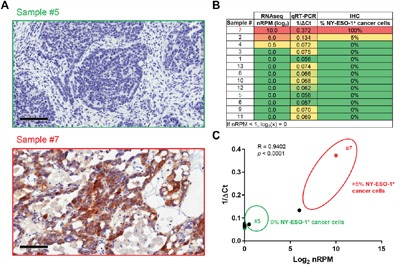
Validation of the Immune Advance assay on NY-ESO-1 **A-C**. Formalin-fixed paraffin embedded (FFPE) samples from 13 ovarian cancer patients were sectioned and processed for immunohistochemical assessment of NY-ESO-1 expression, RNA extraction followed by targeted RNAseq on a panel of immunological transcripts or qRT-PCR-assisted quantification of *CTAG1B* (NY-ESO-1-coding) mRNA levels (*GAPDH* expression was monitored as internal reference). A. Representative images of NY-ESO-1 expression levels as assessed by immunohistochemistry (IHC) on samples #5 and #7. Scale bars = 100 μm. B. Summary of results from RNAseq, qRT-PCR and IHC. C. Correlation of RNAseq (log_2_-transformed normalized reads per million, nRPM) and qRT-PCR (1/ΔCt) results. Samples #5 and #7 are indicated; circles delineate samples with negative (0%) or positive (≥5%) NY-ESO-1 staining by IHC. Pearson correlation coefficient (R) and *p* value are reported.

**Figure 3:**
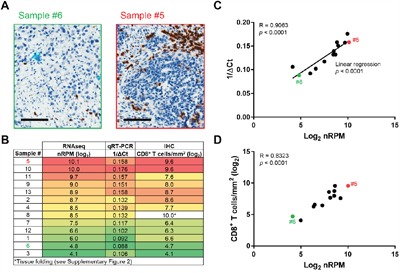
Validation of the Immune Advance assay on CD8 **A-D**. Formalin-fixed paraffin embedded (FFPE) samples from 13 ovarian cancer patients were sectioned and processed for immunohistochemical assessment of CD8 expression, RNA extraction followed by targeted RNAseq on a panel of immunological transcripts or qRT-PCR-assisted quantification of *CD8* mRNA levels (*GAPDH* expression was monitored as internal reference). A. Representative images of CD8^+^ T-cell infiltration as assessed by immunohistochemistry (IHC) on samples #5 and #6. Scale bars = 100 μm. B. Summary of results from RNAseq, qRT-PCR and IHC. C. Correlation of RNAseq (log_2_-transformed normalized reads per million, nRPM) and qRT-PCR (1/ΔCt) results. Samples #5 and #6 are indicated. Linear regression trend, Pearson correlation coefficient (R) and *p* value are reported. D. Correlation of RNAseq (log_2_-transformed normalized reads per million, nRPM) and IHC (CD8^+^ T cells/mm^2^). Samples #5 and #6 are indicated; Pearson correlation coefficient (R) and *p* value are reported. See also Supplementary Figure S2.

**Figure 4:**
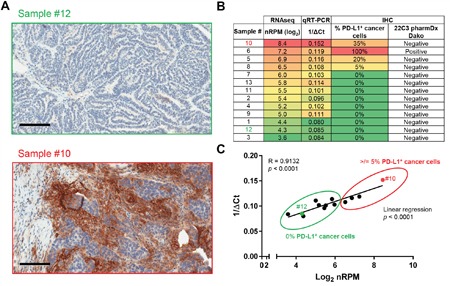
Validation of the Immune Advance assay on PD-L1 **A-C**. Formalin-fixed paraffin embedded (FFPE) samples from 13 ovarian cancer patients were sectioned and processed for immunohistochemical assessment of PD-L1 expression, RNA extraction followed by targeted RNAseq on a panel of immunological transcripts or qRT-PCR-assisted quantification of *CD274* (PD-L1-coding) mRNA levels (*GAPDH* expression was monitored as internal reference). A. Representative images of PD-L1 expression as assessed by immunohistochemistry (IHC) on samples #10 and #12. Scale bars = 100 μm. B. Summary of results from RNAseq, qRT-PCR and IHC. IHC scoring as per Dako HC223 pharmDx guidelines is indicated. C. Correlation of RNAseq (log_2_-transformed normalized reads per million, nRPM) and qRT-PCR (1/ΔCt) results. Samples #10 and #12 are indicated; circles delineate samples with negative (0%) or positive (≥5%) PD-L1 staining by IHC. Linear regression trend, Pearson correlation coefficient (R) and *p* value are reported.

To obtain insights into the potential clinical application of the Immune Advance assay we compared nRPM values (as obtained by targeted RNAseq) with ΔCt values (as obtained by qRT-PCR) for NY-ESO-1, CD8 and PD-L1, finding robust correlation coefficients of 0.9402 (*p* < 0.0001), 0.9063 (*p* < 0.0001) and 0.9132 (*p* < 0.0001), respectively (Figures 2-4). The immunohistochemical assessment of NY-ESO-1 levels identified 2/14 (14%) positive samples, with sample #2 expressing NY-ESO-1 in 5% of neoplastic cells, and sample #7 in its totality. nRPM values also highlighted a similar binary distribution of positive *versus* negative samples, and RNAseq results correlated with both qRT-PCR and IHC findings, although the analysis was limited by the presence of only two positive specimens (Figure 2A-2C).

The expression of CD8 as determined by RNAseq and IHC was regularly distributed across specimens, which facilitated correlation studies. For example, while sample #5 exhibited 761 CD8^+^ T cells/mm^2^, sample #6 only exhibited 26 CD8^+^ T cells/mm^2^. Log_2_-transformed nRPM and 1/ΔCt values for CD8 linearly correlated with each other over a continuous range (4.1-10.1 log_2_ nRPM, 0.88-0.176 1/ΔCt) with the standalone exception of sample #8 (Figure 3A-3C). This specimen was incorrectly scored as containing 1019 CD8^+^ T cells/mm^2^ owing to a section folding artifact (Supplementary Figure S2). Excluding sample #8, the number of log_2_-transformed CD8^+^ T cells/mm^2^ significantly correlated with log_2_-transformed nRPM values (*p* < 0.0001) (Figure 3D).

Finally, four samples exhibited some degree of membranous PD-L1 staining in 5%-100% neoplastic cells. These specimens (namely, samples #5, #6, #8 and #10) also exhibited high nRPM values. Interestingly, the specimen with the highest amount of PD-L1-encoding RNA as per the Immune Advance assay, namely sample #10, only contained 35% PD-L1^+^ neoplastic cells. It is important to recognize that sample #10 would be considered a negative result according to the HC223 pharmDx scoring guidelines. Log_2_-transformed nRPM and 1/ΔCt values for PD-L1 exhibited robust linear correlation, implying that the expression of this clinically-relevant biomarker is not binary like that of NY-ESO-1, but rather continuous such as that of CD8 (Figure 4A-4C).

## DISCUSSION

To assess the usefulness of RNAseq in profiling a panel of immune transcripts on FFPE *versus* FF samples, we utilized a stringent quality control process for tumor heterogeneity that is unique to our study and to the best of our knowledge has never been applied before [34–38]. Tumors were halved as FF and FFPE mirror images with the cut section of each half used for analysis. Basic quality control factors for RNAseq such as mapped reads were consistent for FFPE and FF samples. Paired samples correlated with coefficients ranging from 0.837 to 0.969. Moreover, as proof of principle, results from RNAseq and qRT-PCR were highly concordant for NY-ESO-1, CD8, and PD-L1. These findings establish the feasibility of measuring a panel of immune transcripts by RNAseq on FFPE samples to develop a biomarker that predict clinical response to ICBs.

Our study also demonstrates the usefulness of a targeted RNAseq panel to replace immunohistochemical markers including CD8 for cytotoxic T lymphocytes, and PD-L1 as predictors of response to ICBs. While there have been numerous publications on the “immunoscore” as a prognostic biomarker for cancer patients [39–41], several obstacles prevent implementation of an IHC-based approach into clinical routine [42]. Moreover, there is disagreement on the definition of a “high” versus a “low” TIL score in absolute terms, as most publications refer to one or a few private or public patient cohorts wherein stratification is based on median values [30, 31]. Despite a small sample size in our study, our results support the notion that CD8^+^ T cells can be quantified and their number linearly correlates with *CD8* mRNA expression, as determined by RNAseq. Moreover, our RNAseq data support the notion that PD-L1 expression is continuous rather than binary, in contrast with IHC results. Whether there is a post-translational mechanism that operates to control PD-L1 exposure in a binary manner remains to be determined. If this is not the case, the test currently employed in the clinic to measure PD-L1 expression may lack sensitivity and accuracy, which would have a negative impact on patient selection of immunotherapy with ICBs.

Of note, some tumors that stain positively for PD-L1 by IHC (including melanoma, NSCLC, renal cell carcinoma, colorectal carcinoma, and castration-resistant prostate cancer) are insensitive to ICBs, suggesting that PD-L1 alone is not a reliable predictor of clinical response [43]. Simultaneously analyzing multiple immunological biomarkers with RNAseq could improve this situation and allow for the identification of a gene signature that reliably identifies patients who will respond to immunotherapy with ICBs. In this study, we were not able to correlate RNAseq data with clinical outcome, owing to the type of specimens we employed (ovarian cancer patients do not receive ICBs as part of the clinical routine). Moreover, our study involved a limited number of patients affected by a single type of tumor, calling for validation experiments in larger and more heterogeneous patient cohorts. Irrespective of these caveats, we did identify a subset of immune transcripts that were co-expressed with PD-L1, and we are evaluating these potential biomarkers in cohorts of melanoma, NSCLC and renal carcinoma patients who receive ICBs as part of their treatment. The road to predicting clinical response to ICBs appears to be more complex than the assessment of a single biomarker like PD-L1. Further experimental and clinical validation of the Immune Advance assay is underway to obtain a robust method for simultaneously measuring the expression of multiple immune transcripts from single FFPE samples. We surmise this may pave the way to improve patient selection for immunotherapy with ICBs.

## MATERIALS AND METHODS

### Patients and specimens

All patients referred to in this report were diagnosed with ovarian cancer and treated at Roswell Park Cancer Institute (RPCI, Buffalo, NY, US). The RPCI institutional board gave explicit approval to the study, and all samples were obtained upon informed consent under an institutional protocol for tissue collection. To control for tumor heterogeneity in an effort to minimize biological variability, freshly procured remnant tissue was sectioned into two approximate halves, one of which was processed as FF in Optimal Cutting Temperature compound, and the other one fixed in formalin and processed as per standard clinical practices. Each half was marked with ink across the surface to maintain original tissue orientation and mounted on slides faced side up (Figure 1). Sections from the mirroring surfaces of both FF and FFPE blocks were cut and stained with hematoxylin and eosin quality review by a qualified pathologist. Additional serial sections were cut for RNA extraction and IHC. A total of 14 matched FFPE/FF pairs corresponding to 28 samples were collected.

### RNA extraction

RNA was extracted from FFPE tissues using the truXTRAC™ FFPE RNA Kit (Covaris), as per manufacturer's instructions with modifications. Briefly, lysates from partially lysed tissue samples were processed immediately for RNA extraction. The truXTRAC™ FFPE RNA Kit is designed for use with the Adaptive Focused Acoustics AFA™ process. Standard de-crosslinking and column purification steps were performed to remove proteins and other cellular components prior to RNA elution in water. RNA was extracted from FF tissues using the AllPrep DNA/RNA Mini Kit (Qiagen), as per manufacturer's instructions. RNA was quantified by means of the Qubit RNA HS Assay Kit (Thermo Fisher Scientific).

### RNAseq library preparation, quantification, pooling and sequencing

Oncomine™ Immune Response Research Assay libraries were prepared using the Ion AmpliSeq™ targeted sequencing technology (Thermo Fisher Scientific), as per manufacturer's instructions. The Assay is a 395 gene panel focused on diverse immunological processes including TCRS, tumor infiltration by immune cells, and other key immune functions (Supplementary Table S3). Briefly, 10 ng RNA was reverse transcribed into cDNA (25 °C, 10 min; 42 °C, 60 min; 85 °C, 5 min; 4 °C, hold) and targets were amplified (99 °C, 2 min; 99 °C, 15 seconds, 60 °C, 4 min, 19X; 10 °C, hold) with a multiplex immune response primer pool targeting 395 genes. Amplicons were partially digested using the FuPa Reagent (50 °C, 10 min; 55 °C, 10 min; 60 °C, 20 min; 10 °C, hold for up to one hour). Barcode adapters were ligated to partially digested amplicons (22 °C, 30 min; 72 °C, 10 min; 10 °C, hold for up to one hour) and purified. Libraries were quantified using the Ion Library Quantification Kit (Applied Biosystems by Life Technologies), as per manufacturer's instructions. Up to 20 libraries normalized to 50pM were pooled in equal molar amounts prior to enrichment and template preparation using the Ion Chef™ system (Thermo Fisher Scientific). 200-bp sequencing was performed on the Ion Proton™ P1v3 chip (Thermo Fisher Scientific) to obtain 2-3M reads per sample. Absolute digital gene expression counts and nRPM values were generated using the Torrent Suite software (v5.0.2) and the immuneResponseRNA plugin (both from Thermo Fisher Scientific).

### Gene expression normalization

A baseline expression profile for 10 endogenous control genes was established based on average RPM counts from the internal control sample NA12878 across eleven sequencing runs. Following determination of baseline expression levels, test samples were normalized based on the formula f(*i*) = x(*i*) / p(*i*), in which the *i*-th endogenous control represents the fold change f(*i*) of the raw read count x(*i*) over the above-mentioned baseline profile p(*i*). The median of fold changes from all these controls was then determined as F = median (f(*i*) | *I* = 1,…10). This value was further used to normalize RPM counts for all genes in the sample according to the formula x' (*i*) = x(*i*) / F, where x(*i*) is the raw read count of the *i*-th gene and x' (*i*) is the normalized expression (nRPM) value to be used for downstream analysis. Finally, nRPM values were log_2_-transformed.

### Immunohistochemistry

A 5μm thick whole section from each FFPE sample was stained with antibodies specific for NY-ESO-1 (E978, Santa Cruz Biotechnology), PD-L1 (22C3 pharmDx, Dako), and CD8 (C8/144B, Dako), according to standard procedures. NY-ESO-1 and PD-L1 expression was evaluated by a board-certified pathologist who interpreted the staining as positive or negative. For NY-ESO-1, a positive sample was defined by moderate to strong cytoplasmic staining with membranous accentuation that is distinct from background in at least 5% of neoplastic cells, while a negative sample was defined by staining in <5% of neoplastic cells. For PD-L1, a positive sample was defined as per FDA-approved guidelines as partial or complete cell membrane staining (≥ 1+) in ≥ 50% of viable tumor cells, while a negative samples was defined by any membranous staining in less than 50% of neoplastic cells. CD8^+^ T lymphocytes were stained and scored using the Aperio Scanscope (Aperio Technologies, Inc.), based on 20X bright-field optical microscopy. Images were analyzed using Spectrum (Aperio Technologies, Inc.) and the number of CD8^+^ T lymphocytes per square millimeter was counted.

### Quantitative RT-PCR (qRT-PCR)

Ten ng RNA was reverse-transcribed (25 °C, 10 min, 37 °C, 120 min; 85 °C 5 min; 4 °C, hold), amplified (50 °C, 2 min, 95 °C, 10 min, 95 °C, 15 seconds, 60 °C, 1 min, 40X), and ΔCt was determined using TaqMan Gene Expression Assays specific for *CTAG1B/1A* (NY-ESO-1), *CD274* (PD-L1) and *CD8* (Thermo Fisher Scientific) on the QuantStudio 7 Real-Time PCR System (Thermo Fisher Scientific). Glyceraldehyde-3-phosphate dehydrogenase (*GAPDH*) was employed as reference transcript. ΔCt values are depicted as 1/ΔCt.

### Statistical analysis

Correlation coefficients were calculated according to the Pearson method. *p* values < 0.05 were considered statistically significant. All statistical analyses were conducted on Prism 7 (GraphPad Software).

## SUPPLEMENTARY MATERIALS FIGURES AND TABLES








