# Supplementary material for: Robust detection of immune transcripts in FFPE samples using targeted RNA sequencing
Source: Oncotarget. 2016 Nov 29;8(2):3197–205. doi: 10.18632/oncotarget.13691 (PMC5356875; doi:10.18632/oncotarget.13691)
Supplement: Supplementary file 1 [file oncotarget-08-3197-s001.pdf]

Robust detection of immune transcripts in FFPE samples using targeted RNA sequencing

SUPPLEMENTARY DATA

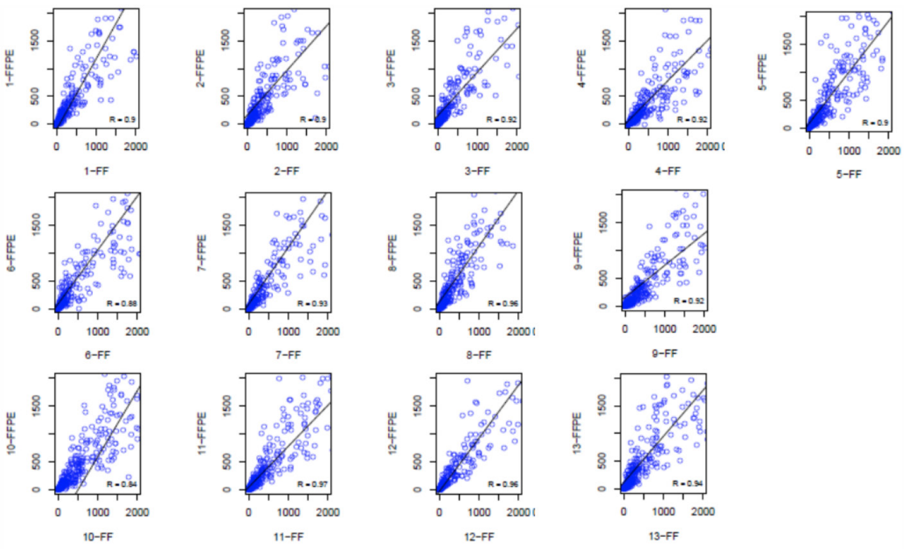

Supplementary Figure S1: Correlation of RNAseq results in FFPE and FF samples. Scatter plots comparing RNAseq results on each of 13 matched FFPE and FF sample pairs from ovarian cancer patients.

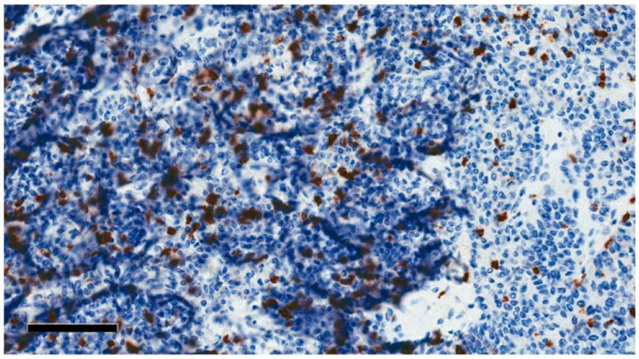

Supplementary Figure S2: Immunohistochemical assessment of $CD8^+$ T-cell infiltration in sample #8. The FFPE sample from patient #8 was processed for the immunohistochemical quantification of CD8 expression followed by automated image analysis for the assessment of $CD8^+$ T-cell infiltration. Manual review of the sample demonstrated that IHC results (a spuriously high value of 1,019 $CD8^+$ T cells/mm^2) did not match RNAseq results on the same specimen (suggesting moderate $CD8^+$ T-cell infiltration) owing to a tissue folding artifact. Scale bar = 100 µm. See also Figure 3.

Supplementary Table S1: Metrics for the runs used in this study. Run metrics were extracted from Torrent Suite™ summary report based on alignment against the IO panel reference v31 (20160614) using target region BED version IO panel v3 (20160720)

Supplementary File 1

Supplementary Table S2: RNAseq sample metrics. Sequence reads were aligned to the IO panel reference v31 (20160614) in Torrent Suite™ using the built-in tmap program. Following alignment, the coverageAnalysis (v5.0.4.0) plugin was employed to determine the number of mapped reads and on target reads (%)

Supplementary File 2

Supplementary Table S3: RNAseq panel composition. Gene symbol, alias, Entrez ID, gene name, and main immunological functions are reported

Supplementary File 3
